# Investigation of mechanical and thermal behavior of fiber-reinforced silica xerogel composites

**DOI:** 10.1371/journal.pone.0303293

**Published:** 2024-06-12

**Authors:** Muhammad Ammar, Aneela Wakeel, Muhammad Ali Nasir, Muhammad Zubair

**Affiliations:** 1 Mechanical Engineering Department, University of Engineering and Technology, Taxila, Pakistan; 2 Department of Mechanical & Nuclear Engineering, University of Sharjah, Sharjah, UAE; Universidad Tecnica de Ambato, ECUADOR

## Abstract

Silica aerogels or xerogels are renowned dried gels with low density, high surface area, higher porosity, and better thermal stability which makes it suitable for aerospace, light weight structures, thermal insulation, and hydrophobic coatings. But brittle behaviour, low mechanical strength, and high manufacturing cost restrict its usage. Recently, the addition of various fibres like glass or carbon fiber is one of the best reinforcement methods to minimize the brittle behaviour. Supercritical drying technique usually used to develop aerogel that is expensive and difficult to produce in bulk quantities. Higher cost obstacle can be tackled by applying ambient pressure drying technique to develop xerogel. But researcher observed cracks in samples prepared through the ambient pressure drying technique is still a major shortcoming. The aim of this study is to systematically analyze the influence of silica gel fiber reinforcement on silica xerogels, encompassing morphology, mechanics, thermal behaviour, compression test, and thermogravimetric characteristics. The research used a low-cost precursor named Tetraethyl orthosilicate to synthesize low-cost composite Silica xerogel and glass and carbon fiber added to provide strength and flexibility to the overall composite. Silica gel works as binder in strengthening the xerogel network. The investigation employs scanning electron microscopy (SEM) to examine the morphology of the composites, Fourier Transform Infrared (FTIR) analysis to affirm hydrophobic characteristics, compression tests to assess mechanical strength, and thermogravimetric tests to study weight loss under different conditions. SEM results reveals that glass fibers exhibit lower adhesion to the xerogel network compared to carbon fibers. FTIR analysis confirms the hydrophobicity of the composite silica xerogel. Compression tests showed that, under a 48% strain rate, the carbon fiber composite demonstrates superior compressive stress endurance. Thermogravimetric tests revealed a 1% lower weight loss for the carbon fiber composite compared to the glass fiber composite. This work concludes that glass and carbon fiber together with silica gel particles successfully facilitated in developing flexible, less costly, hydrophobic, and crack-free silica xerogel composites by APD. These advancements have the potential to drive innovations in material science and technology across diverse industries.

## Introduction

Silica Aerogel (SA) captivates researchers with its nanoscale structure, forming a porous network of interconnected silica particles encapsulating air or gases. Its notable attributes, including low density, expansive surface area, low thermal conductivity, high porosity, and remarkable thermal stability [1], position it as a material of great potential for diverse applications. However, challenges such as brittleness and high production costs have restricted its use, particularly in thermal insulation [1].

The synthesis of SA unfolds through Hydrolysis, Gelation, Aging, and Drying [2]. Hydrolysis controls the reaction speed by initiating the interaction between silicon alkoxide and water under acidic conditions. Gelation produces linear or branched chains of silica particles, and Aging strengthens the alcogel structure while purifying it. Subsequent Drying removes excessive solvent, resulting in a highly porous aerogel structure predominantly composed of air [2].

Various drying techniques, including supercritical drying (SCD), freeze-drying (FD), and ambient pressure drying (APD) [3–5], have been explored. Despite its exceptional porosity, SCD faces challenges due to high costs and difficulty in maintaining supercritical conditions [6]. Alternative methods such as freeze-drying and ambient pressure drying are considered for their economic viability. However, challenges like extended aging times, structural issues, and substantial shrinkage during APD require careful consideration and surface treatment methods to mitigate these limitations [7,8].

Recent advancements in modifying gel materials have broadened the horizon of SA research. Innovations, such as introducing inorganic ceramic fibers by Xiaoguang Yang et al., enhance compressive strength, particularly under increased aerogel temperatures [9]. Zhi Li et al.’s exploration of adding aramid fibers reveals improvements in porosity, thermal conductivity, and compressive strength—a breakthrough in thermal insulation applications [10]. Further analyses by Xiaobing Tang et al. show that an increased percentage of silica nanowires substantially improves compressive strength and slightly alters thermal conductivity, showcasing the morphological compatibility of the composite aerogel [11].

Subramaniam Iswar et.al. prepared silica aerogel with the help of Polyethoxydisiloxane, TEOS, and ethanol, followed by surface modification using hexamethyldisiloxane, concentrated hydrochloric acid, and ethanol. Both APD and SCD techniques are used for drying aerogels. SEM images show typical silica aerogel morphologies, including mesopores surrounded by secondary particle agglomeration. The density measured was 0.136 g/cm^3^ for the samples aged for 24 hours at 55°C temperature. Compression test and thermal stability were not done in this study [12]. In another study, TEOS with methanol, oxalic acid, and ammonium fluoride was used for the preparation of wet gel after that necessary surface modification using hexamethyldisilazane was utilized. Silica nanoparticles were doped in the silica matrix during the gelation stage. APD technique was employed. SEM showed less porous structure than silica aerogel with no SiO_2_ addition. The three-dimensional network structure of interlinked silica particles existed with good dispersion of SiO_2_ in the composite aerogel. The density evaluated for the composite aerogel was 0.350 g/cm^3^. A compression test was not done by this study while thermally stable up to 369°C with a weight loss of 7% [13]. Sameera Shafi, et.al synthesized silica aerogel with silica gel and GF reinforcements by using SCD. TEOS together with ethanol, deionized water, hydrochloric acid, and ammonium hydroxide was used for the preparation of wet gel. Silica aerogel was reinforced with different percentages of silica gel with no specified glass fibers quantity used. As, the samples were prepared using the SCD technique. No surface modification process was done during production. The fiber addition improved the mechanical strength of the matrix. Silica gel helped in improving the adhesion property of silica particles with glass fiber. The SEM images showed randomly distributed glass fibers attached with the silica matrix enclosed with silica gel. As the silica gel weight percentages increased, the porosity of the structure decreased with decreased void spaces. The density of 0.231 g/cm^3^ was computed for this composite silica aerogel. A compression test was done to analyze the mechanical properties. The maximum compressive stress was observed to be 1000 kPa. The thermal gravimetric analysis was not examined in this study [14]. Silica aerogel with Carbon Fiber reinforcement was prepared by Agnieszka Slosarczyk using the APD technique. Water glass was used as a precursor with citric acid to promote hydrolysis and methanol as a solvent. Necessary surface modification was done to help in resisting cracks formation during APD by using TMCS and n-hexane solution. SEM image showed well-organized carbon fibers with silica particles to enhance the strength of composite silica aerogel. Void spaces were present in the structure with pores in the silica aerogel matrix. The calculated sample density was 0.199 g/cm^3^. The compression modulus was estimated to be 54 kPa while samples were thermally stable up to 400°C [15].

Despite these advancements, a crucial research gap persists, motivating the current study. The brittle nature of SA during the drying process necessitates reinforcements to enhance mechanical strength and reduce the risk of cracking. Leveraging the ambient pressure drying technique, this study incorporates silica gel, glass, and carbon fibers into the xerogel network, aiming to increase the mechanical strength. This addition enables the xerogel structure to withstand compressive stresses and capillary pressure forces during drying. Surface modification, utilizing highly reactive trimethylchlorosilane in conjunction with n-hexane, further enhances hydrophobicity. The overarching objective is to significantly contribute to thermal insulation applications through the development of Silica xerogel composites, addressing brittleness concerns and enhancing overall sustainability and application potential.

## Materials and method/methodology

### Materials

In the preparation of Silica Xerogel Composites, Tetraethyl orthosilicate is used as a precursor having a density of 940 kg/m^3^ and molar mass of 208.33 g/mol. Trimethylchlorosilane is used as a surface modification chemical having a density of 856 kg/m^3^ and a molar mass of 108.64 g/mol. Both are purchased from Daejung Chemicals & Metals Co Ltd, Korea. Deionized water having a density of 997 kg/m^3^ and molar mass of 18.015 g/mol is purchased from Vitro Diagnostics Laboratories, Islamabad, Pakistan. Ethanol, hydrofluoric acid, and ammonium hydroxide have a density of 789 kg/m^3^, 1.15 kg/m3, 880 kg/m^3^, and molar mass of 46.07 g/mol, 20.0063 g/mol, 35.04 g/mol respectively, are purchased from Sigma Aldrich Company, Germany. Silica gel (technical grade, pore size 60 Å, 70–230 mesh, 63–200 μm) was purchased from Sigma Aldrich. Glass fiber (Density 2500 kg/m^3^, Diameter 8–10 μm), Carbon fiber (Density 2000 kg/m^3^, Diameter 7–10 μm) are purchased from the Chinese manufacturer. The materials involved in the synthesis of composite silica xerogel are utilized without supplementary purification.

### Synthesis of composite silica xerogel

The synthesis of silica xerogel undergoes meticulous steps encompassing Hydrolysis, Gelation, Aging, and Drying. Hydrolysis involves the precise reaction of silicon alkoxide with water under acidic conditions to regulate the reaction speed. The tetraethyl orthosilicate, ethanol, and deionized water are blended in a volume ratio of 3:6.5:1 with continuous mixing at 1000 rpm using a magnetic stirrer for an hour. The solution is then treated with some drops of hydrofluoric acid to reduce the pH to 3 before being agitated for another 10 minutes. Following that, 0.05 weight percentage of silica gel is added together with 0.25M ammonium hydroxide to raise the pH to 6 and agitated for 5 minutes to obtain a well-dispersed solution with a hazy appearance. After the hydrolysis process, the pouring procedure begins to commence the gelation process in the moulds with randomly arranged carbon and glass fibers. The wet gel was left in the moulds for an hour to increase its strength. Gelation facilitates the formation of structured chains of silica particles, while Aging strengthens the alcogel structure and purifies it from impurities. The solvent exchanging operation is then conducted. Wet gels are immersed in ethanol and n-hexane for 1 day each. For 24 hours, the surface of wet gel is altered with trimethylchlorosilane and n-hexane in a 1:3 volume ratio. After that, the wet gel is rinsed using n-hexane and the drying phase starts. The subsequent Drying phase removes excess solvent, yielding a highly porous aerogel structure primarily composed of air [2].

Critical to the study, glass and carbon fibers are procured with precision in terms of dimensions and composition. The composite mixture, now incorporating reinforcing fibers, is cast into molds of precise dimensions. Glass fibers, recognized for their durability and transparency, and carbon fibers with high tensile strength are chosen deliberately. Controlled curing conditions are employed to facilitate the solidification of the composite materials.

The wet gels are dried in an oven for three hours with a gradual change of temperature from 60°C to 100°C at atmospheric pressure to obtain composite SX. This step is crucial to achieving consistent and reproducible results, as variations in curing conditions could introduce disparities in the mechanical and thermal properties of the composites. Predetermined percentages of glass and carbon fibers are integrated into the silica xerogel matrix to create composite samples with varying fiber content. This deliberate integration aims to strike an optimal balance between reinforcement and the intrinsic properties of the silica xerogel.

**Fig 1:**
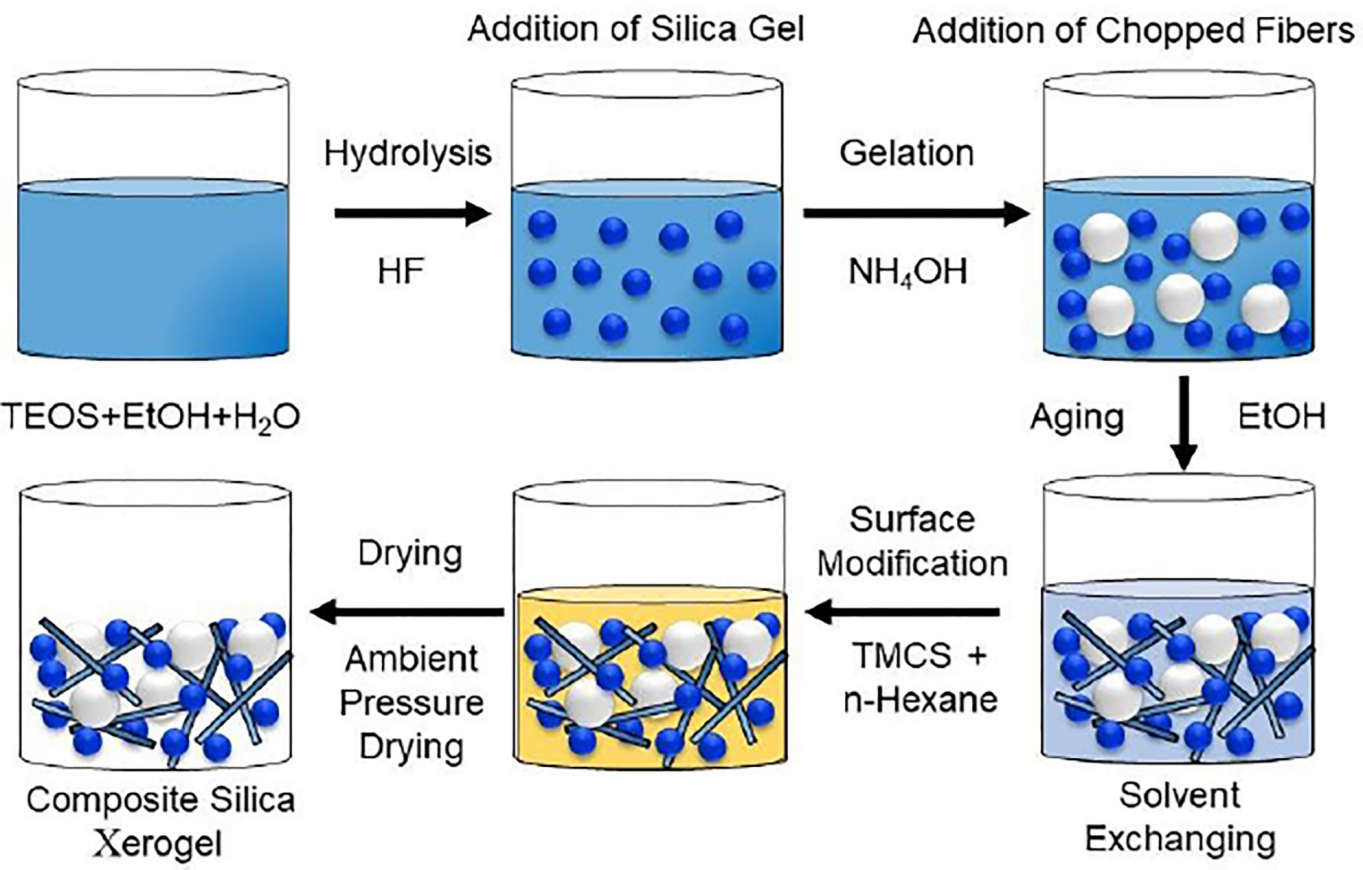
Process diagram of synthesis of composite silica xerogel.

Fig 1 depicts the step-by-step process followed during the synthesis of composite silica xerogel.

Four different types of samples were synthesized. The samples are described as follows:

S1 sample: Silica Xerogel with no additive and no fibers.

S2 sample: Composite Silica Xerogel containing 0.5% silica gel as an additive.

S3 sample: Composite Silica Xerogel with Glass Fiber reinforcement and 0.5% silica gel.

S4 sample: Composite Silica Xerogel with Carbon Fiber reinforcement and 0.5% silica gel.

### Characterization

#### Density measurements

Density measurements are conducted to evaluate the mass per unit volume of the composite silica xerogels. This metric provides essential information regarding the material’s compactness and contributes to a comprehensive understanding of its physical properties. Composite SX densities were calculated by conventional formula i.e., mass to volume ratio.

#### Microstructural analysis

Microstructural analysis employs SEM to examine the fractured surfaces of composite samples post-mechanical testing. This detailed examination allows for the visualization of the distribution and alignment of reinforcing fibers within the silica xerogel matrix. SEM imaging provides crucial insights into the microstructure, influencing the material’s mechanical properties. The Quattro Environmental SEM (USA) was used to investigate morphological properties. For the sample preparation of the SEM samples, Desk V Hp Series (USA) was used to deposit a thin layer of gold on the sample’s surface.

#### Fourier Transform Infrared (FTIR) analysis

FTIR testing is incorporated to analyze the chemical composition of the composites. Spectra are obtained, allowing for the identification of functional groups and molecular structures within the material. This test provides valuable insights into the interaction between the silica xerogel matrix and reinforcing fibers. The chemical composition (Functional groups) of the samples was examined by Attenuated FTIR spectrum carried out by IRTracer-100 (Japan).

#### Mechanical testing

The compression test aims to assess compressive strength, modulus of elasticity, and deformation behaviour. Axial compression tests are conducted on composite samples using a universal testing machine. Stress-strain curves are meticulously recorded to analyze the material’s response to compressive forces, offering insights into its structural integrity under pressure. The mechanical behaviour of the composite silica xerogels was assessed by using Ultimate Tensile Machine AG-X Plus (Shimadzu AG-X Plus, Japan).

#### Thermogravimetric Analysis (TGA)

TGA examines the thermal stability and degradation temperature of the fiber-reinforced silica xerogel composites. Samples are subjected to controlled heating, and weight changes are recorded. This analysis provides insights into the material’s response to increasing temperatures and its thermal decomposition characteristics. The Thermogravimetric Analyzer TGA-51 (USA) assisted in determining the thermal stability of samples.

## Results and discussion

**Fig 2:**
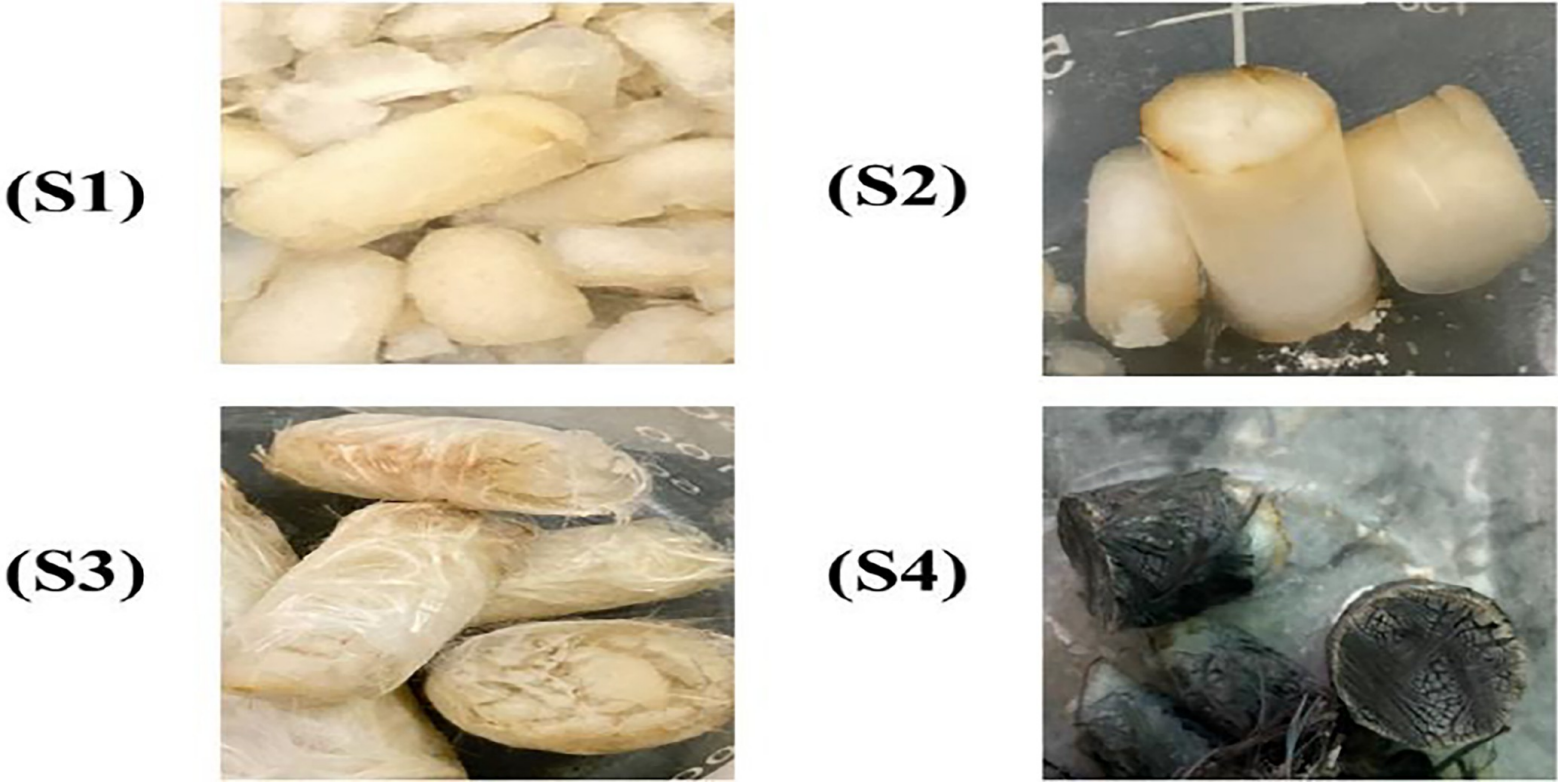
Images of synthesized xerogels. Silica xerogel (S1), Composite silica xerogel (S2), Silica xerogel with glass fibers (S3), Silica xerogel with carbon fiber (S4).

In this study, the silica xerogel, composite silica xerogel, and composite SX with glass and carbon fiber reinforcement were successfully produced by utilizing the APD technology to reduce the cost of production and investigate their influence on xerogel characteristics. Fig 2 depicts the visual representation of silica xerogel synthesized during this work i.e., Silica Xerogel(S1), Composite SX(S2), Composite SX with GF(S3), and Composite SX with CF(S4). The samples were manufactured in a limited number of days, approximately 60 hours. Silica xerogel is comprised of crosslinked silica particles network. But due to cracking during evaporation of solvent, it showed as granules. Silica xerogel composite contained silica particles bridged with silica gel to strengthen its structure. The drying approach used in this study is ambient pressure drying, which is reliable and simple to use, unlike supercritical drying techniques, which have significant pressure and temperature restrictions, resulting in expensive aerogel pricing. The foremost problem of crack formation during APD was minimized with the help of silica gel and fiber inclusion.

### Morphological properties

#### Density

The density of the samples was easily computed by dividing the mass by their volume. The density of SX was found to be 0.1564 g/cm^3^, whereas the density of composite SX was calculated to be 0.2789 g/cm3. The densities of composite SX with GF and CF were 0.1757 g/cm^3^ and 0.1736 g/cm^3^, respectively. The density was slightly affected by the incorporation of glass and carbon fiber together with silica gel inclusion because of the stronger force of attraction between fiber and xerogel matrix results in a compact structure. Previous studies showed that the density of simple SX, composite SX, Composite SX with GF, and CF was observed to be 0.136 g/cm^3^ [12], 0.350 g/cm^3^ [13], 0.231 g/cm^3^ [14], and 0.199 g/cm^3^ [15]. Density of the samples are represented in a well-organized way as shown in Table 1. Additionally, some density values from literature are presented for comparison. This tabular representation provides a clear and concise overview of the density values for the different samples and allows for easy comparison with previous studies.

**Table 1:** Density of silica xerogel samples.

Sample	Density (g/cm^3^)	Density (g/cm^3^) literature
SX	0.1564	0.136 [12]
Composite SX	0.2789	0.350 [13]
Composite SX with GF	0.1757	0.231 [14]
Composite SX with CF	0.1736	0.199 [15]

#### Scanning electron microscope

**Fig 3:**
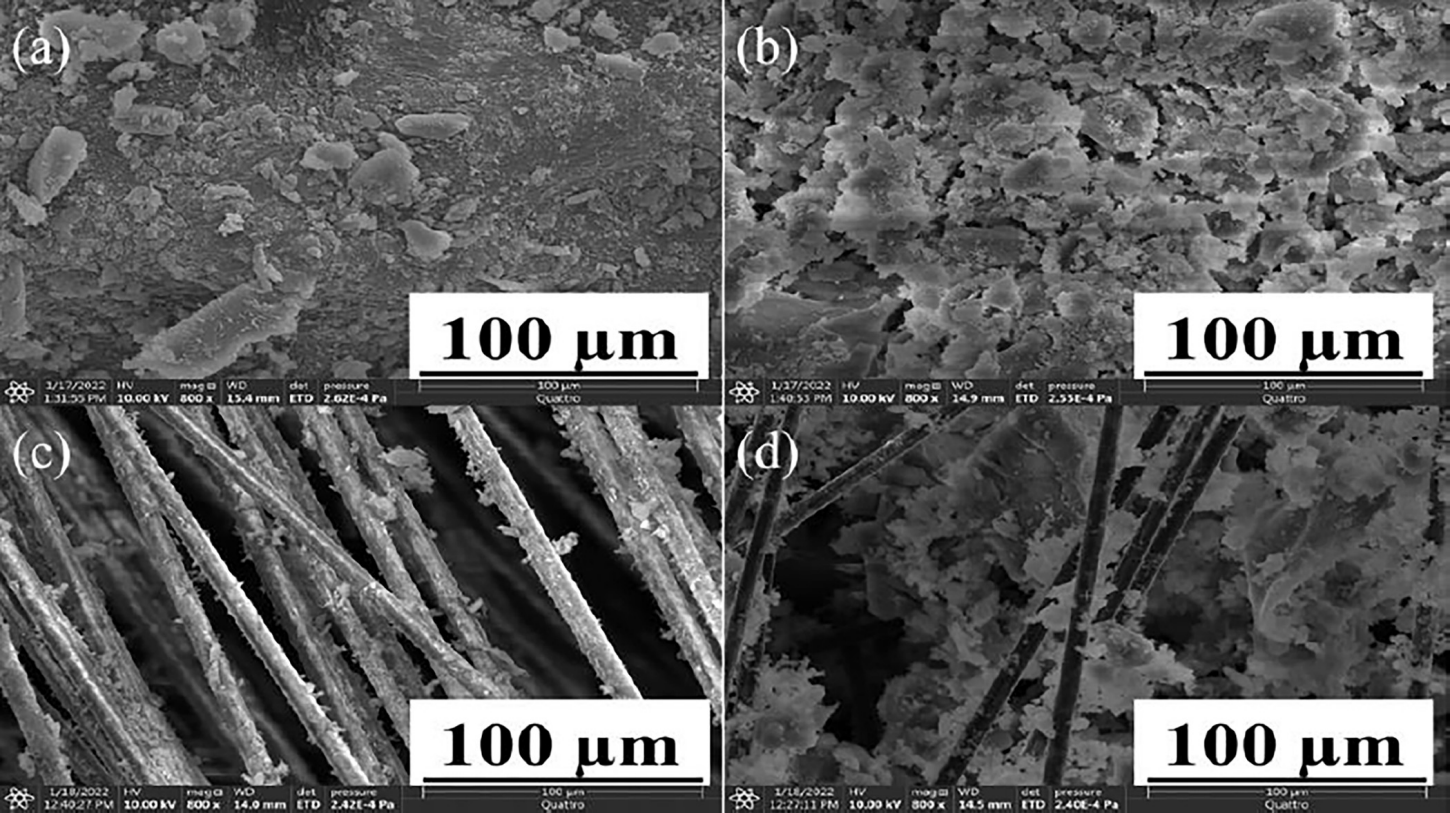
(a) SEM images for silica xerogel (S1) (b) SEM images for composite silica xerogel (S2) (c) SEM images for composite silica xerogel with glass fibers (S3) (d) SEM images for composite silica xerogel with carbon fiber (S4).

In Fig 3(A), silica xerogel’s scanning electron micrograph was taken at a magnification of 800x to analyze the structure of the xerogel formed. The silica xerogel structure collapsed during the drying process because the xerogel network was unable to bear such an extreme capillary pressure caused by the evaporation of solvent. Hence, Fig 3(A) depicted the disintegrated structure comprised of small granular random shapes along with the existence of powdered silica xerogel. The average size of granular is observed to be 24.62 μm. V. V. Ganbavle et.al. found that the aerogel’s surface was very porous, as intended, according to the SEM while synthesizing silica aerogel with methanol as a solvent and continuous shaking during aging [16]. In Fig 3(B), SEM image of composite SX was taken. The addition of silica gel reduced the crack formation and collapsing of the structure during the drying stage thus it behaves as a binder resulting in the strengthening of the xerogel matrix. Fig 3(B) showed a three-dimensional network of silica xerogel comprising of well-dispersed silica gel particles along the xerogel matrix. Few void spaces present in the xerogel can be minimized by increasing the silica gel content. By comparison of the SEM images shown in Fig 3(A) and Fig 3(B), it was found that the addition of silica gel into the wet gel gives strength to the Si-O-Si structure of xerogel upon drying as well as helps in resisting the disintegration of xerogel structure. In-Keun Jung et.al observed that increased silica nanoparticle substitution resulted in a much denser structure because the surface -OH groups and particle size of aerogel were altered without modifying the pH, aging, or drying conditions [13].

In Fig 3(C), SEM image of composite SX with glass fiber was taken. Glass fibers were added to the silica xerogel composite with silica gel addition to enhance the bonding strength of the xerogel matrix with the fiber. The average diameter of the glass fiber is 9.29 μm. The small size of glass fiber gave a high contact surface area to the xerogel so that it can bind firmly with the xerogel matrix. The images demonstrated that the fibers were covered with a limited amount of xerogel structure due to the high concentration of fiber content in the composite SX. Silica gel with glass fibers greatly enhanced the structure of xerogel with no collapsing of structure along with the production of flexible xerogel composite. Void spaces which can be observed in Fig 3(C) can be minimized by increasing the silica gel concentration. Sameera Shafi et.al analysed that the silica aerogel’s 3D nanoporous structure was unaffected by the addition of the GF. Furthermore, the aggregated clusters became denser, and the structural appearance of the particles was substantially transformed when the methyltrimethoxysilane (MTMS) quantity was increased.

In Fig 3(D), carbon fiber was used along with the xerogel and silica gel. The average diameter of fibers determined from SEM images is 7.49 μm. The adhesiveness of the carbon fibers with xerogel matrix was improved with silica gel addition due to low diameter of the carbon fibers which results in high contact surface area as compared with Fig 3(D) containing SEM images of composite SX with GF. Few voids are also present as compared with glass fiber SX composite in Fig 3(C). Agnieszka Slosarczyk analyzed the adhesion at the interface between silica aerogel and carbon fibers. The interaction between the oxidized fiber surface and the hydroxide group present on the silica aerogel surface led to extremely high adherence of aerogel to the fiber surface [15].

#### Fourier transform infrared spectroscopy

**Fig 4:**
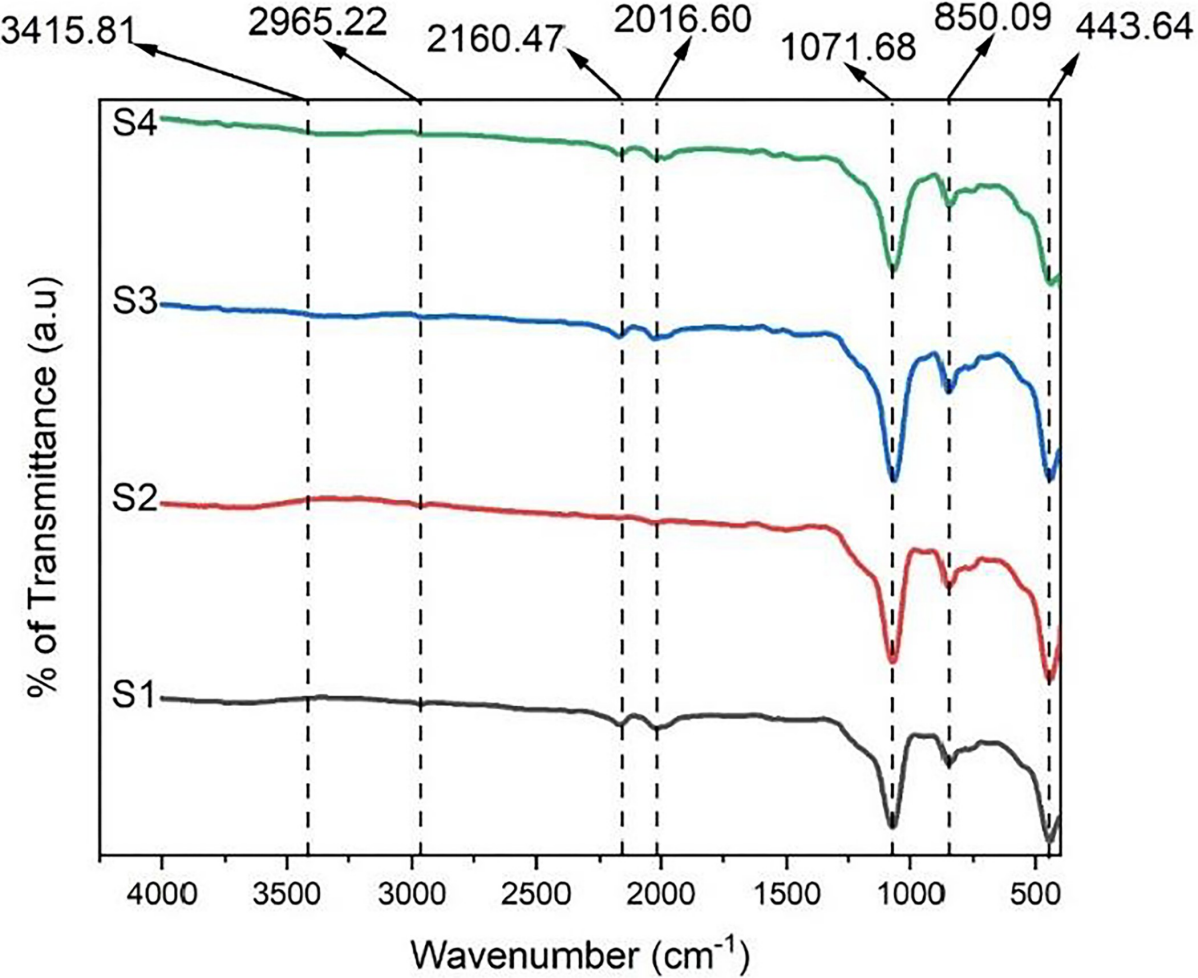
FTIR graph for the synthesized xerogels.

The FTIR analysis was performed to analyze the infrared spectrum of the samples as depicted in Fig 4. The peak observed at 443.64 cm^-1^ illustrates the asymmetric bending vibration of Si-O-Si bonds. The strongest peak at 1071.68 cm^-1^ represents symmetric and asymmetric stretching vibrations of the Si-O-Si bond. This confirms the formation of SiO_2_ bonds containing a network of Si-O-Si structures within our synthesized samples. The peak at 850.09 cm^-1^ corresponds to the presence of the Si-C bond. Peaks at 2160.47 and 2016.60 cm^-1^ are due to the stretching vibrations caused by the occurrence of the C ≡ C bond. The peaks at 2965.22 cm^-1^ depict the presence of a C-H bond. This peak shows the stretching and bending of the C-H bond. No significant peak ranges between 3390–3200 cm^-1^ which shows negligible existence of the Si-OH group [17,18]. The synthesized xerogel samples are truly hydrophobic with the non-existence of peak value that is associated with the -OH group. From the graph, it is concluded that there is no significant difference between the spectra of SX and composite SX which make the xerogel versatile by giving new properties to the material by adding filler and fiber without disturbing the chemical bonds present in the structure. Asep Bayu Dani Nandiyanto et al investigated the data interpretation of FTIR. The summary of functional group and wavenumber were discussed in detail in this research article which helped in determining the chemical bonds present in the samples [19].

### Mechanical testing results

Compression tests were performed on the samples to explore the mechanical characteristics of the composite SX. The test is performed at a speed of 0.1 mm/min. Samples are prepared according to the dimensions in ASTM standard D-695. The length of the specimen is 1 inch and diameter of ½ inch. The compression test cannot be performed for silica xerogel (S1) because of its existence in granular form.

**Fig 5:**
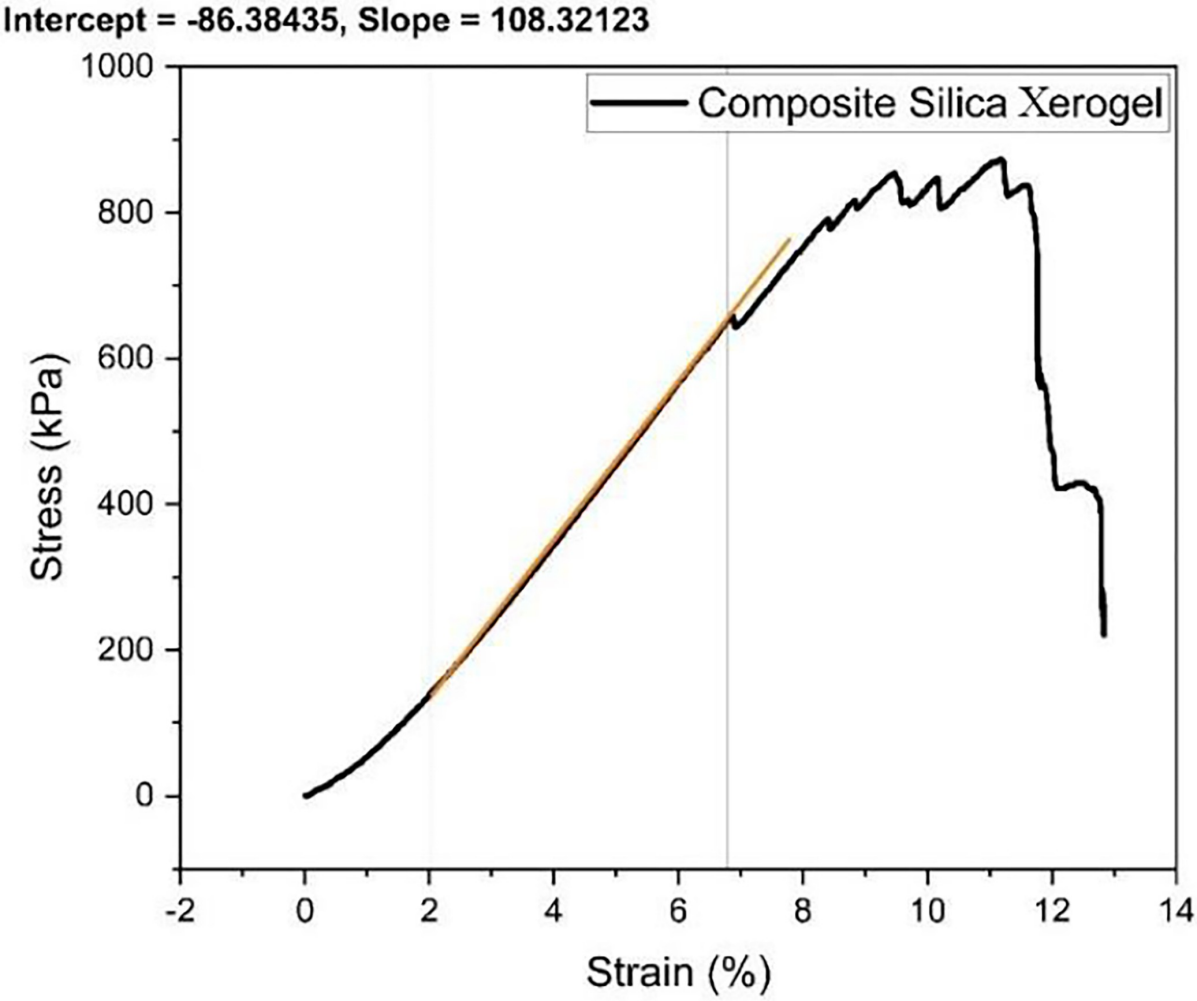
Stress-strain graph for composite silica xerogel (S2).

**Fig 6:**
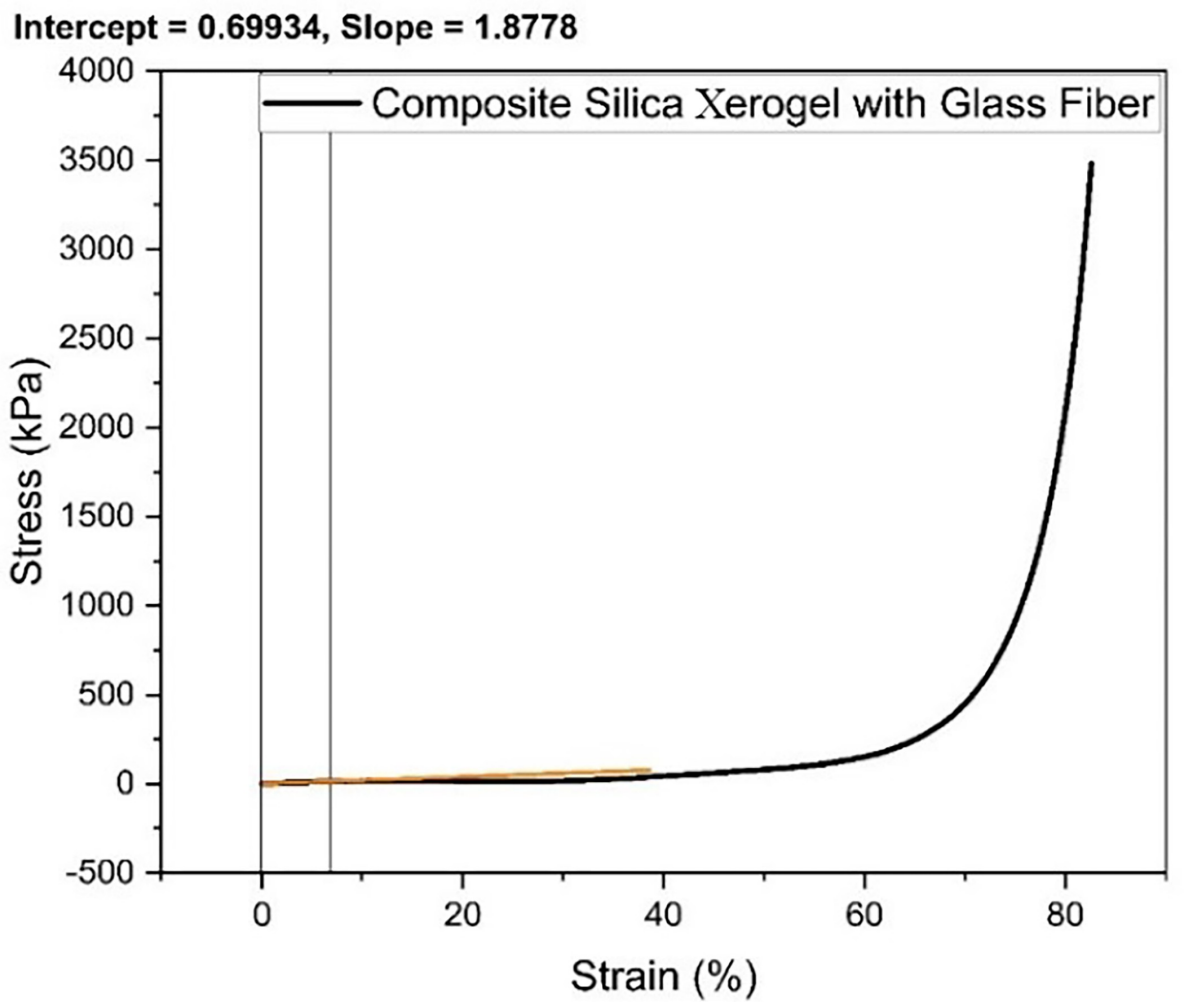
Stress-strain graph for composite silica xerogel with glass fibers (S3).

**Fig 7:**
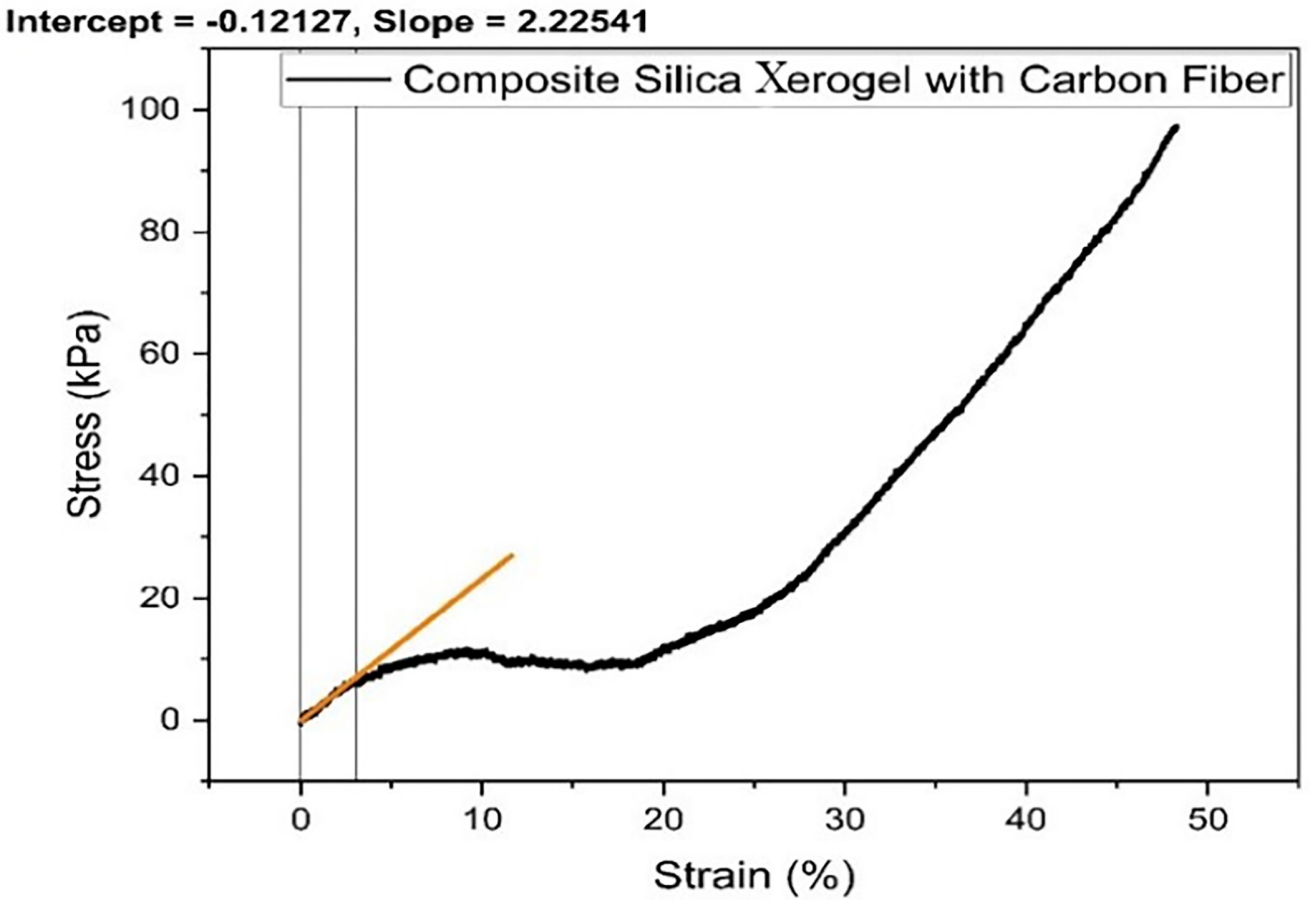
Stress-strain graph for composite silica xerogel with carbon fibers (S4).

Graphs between stress and strain for the samples are shown in Figs 5, 6 and 7. The silica xerogel composite (S2) shows a linear behavior at first then gradually decreased by making a dome shape as shown in Fig 5. The stress is directly proportional to strain from 0 to 7% strain. After that, irregular behavior is observed due to the formation and propagation of cracks. After the linear stage, it starts resisting the applied stress at some place, and on the other side, it promotes crack flow. The compressive modulus of Composite SX was found to be 108.32 kPa, calculated using Origin Pro. The compressive stress of the composite silica xerogel depicts the highest value at 10% strain when compared with other samples due to the addition of silica gel. This shows the brittle nature of the sample with silica gel impregnation as compared with other samples.

Glass fiber addition greatly enhances the compressive strength of the xerogel structure. Firstly, the behavior of the sample is linear, but it begins to increase exponentially after the strain of 60% as shown in Fig 6. The composite SX with GF (S3) sample possessed good deformability up to 82% strain. The stress-strain graph of composite SX with GF is comprised of four stages. The contact stage exists at low strains ranging from 0 to 15% because of the irregular surface contact of the sample with the load cell and fixture. Afterward, the stress and strain increased linearly at strains ranging from 30 to 45% during the linear stage of the stress-strain diagram. In this stage, the large particles of silica gel and the nanopores of silica xerogel served as the primary bearing element. At the same time, the fibers were solely responsible for the stability of the composite SX with GF. During the yielding stage from 45 to 60% strain, the stress grew significantly, and the fibers contributed to the predominant bearing component, which stood distinct from the pure xerogels. Furthermore, during the densification stage, the slope grew substantially. The principal reasons were the collapse of the xerogel and the subsequent densification of the porous structure. Amaya Rege et. al reported same super flexible nature of composites due to fiber and aerogel combination [20]. The compressive modulus of Composite SX with GF was found to be 187.78 kPa, calculated using Origin Pro.

Samples with carbon fiber substitution exhibit a similar trend as composite SX with GF does, but with some extent of linearity along with less deformation up to just 48% as shown in Fig 7. The stress-strain curve of the composite SX with CF (S4) is divided into three stages. The first stage is named as Linear/ Elastic stage in which the stress is directly proportional to the strain and ranges from 0 to 10% strain due to the rough exterior surface of the xerogel in contact with the load cell and fixture and squeezing of nanopores of the silica xerogel present in the sample. After that, the Extra-elastic stage begins which ends up to 20% strain in which the fibers act as a supporting member of the xerogel matrix to bear the compressive stress. Furthermore, the stress-strain curve started rising significantly due to the compacting of the xerogel composite followed up by xerogel network degradation. The compressive modulus of Composite SX with CF was found to be 227.36 kPa, calculated using Origin Pro. Agnieszka Slosarczyk et.al investigated that the xerogel composite with 10% carbon fiber addition had substantially greater compressive strength, and the specimens recovered to their original forms at a 93% rate when the load was removed [15]. The higher number of fibers resulted in a weak structure due to the formation of micropores and inactive fibers which were not well bonded with the silicon structure.

Samples with glass and carbon fiber addition are much more flexible than samples with no fiber addition. Hence, the deformability of silica xerogel with glass fiber reinforcement was noted to be much higher than with carbon fiber reinforcement. Moreover, the carbon and glass fiber addition make the xerogel structure stronger and more flexible, resulting in increased bearing compressive strength at higher strains without collapsing the xerogel structure and retaining its 90% shape originality. Hence, the mechanical properties of the samples were greatly enhanced by the addition of glass and carbon fiber along with the silica addition by increasing the adhesiveness of the fiber with the xerogel network. A summary of the elastic modulus of the samples are described in Table 2.

**Table 2:** Elastic Modulus of the samples.

Samples	Elastic Modulus (kPa)
S1	-
S2	10832.12
S3	187.78
S4	227.36

### Thermal gravimetric analysis

**Fig 8:**
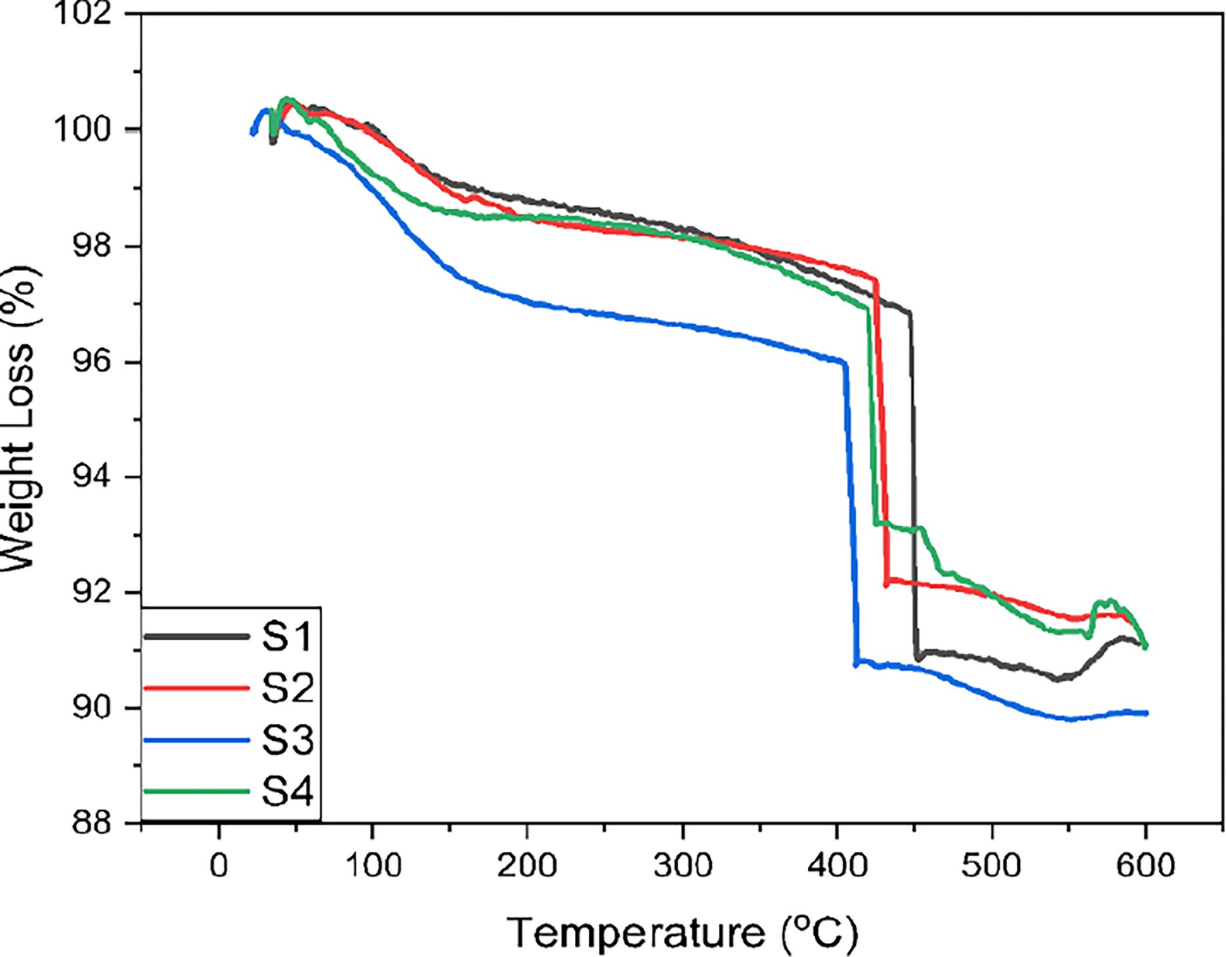
TGA graph for the synthesized xerogels.

Thermal stability analysis, illustrated in Fig 8, offers a detailed examination of the behavior exhibited by silica xerogel samples under various reinforcement procedures, scrutinized up to a temperature of 600°C. The thermal gravimetric analysis (TGA) curve, divided into three distinct phases, unveils critical insights into the material’s thermal response throughout different stages of heat exposure.

Commencing the thermal journey in Part I (Evaporation of Liquid Constituents), the TGA graph captures the initial phase marked by the evaporation of liquid constituents—water and ethanol—present in the xerogel samples. This foundational stage represents the elimination of volatile components, setting the stage for subsequent thermal transformations. Advancing into Part II (Oxidation of Silicon-Methyl Bond), the subsequent stage unfolds the oxidation of the silicon-methyl bond situated on the xerogel samples’ surface. This phase signifies a crucial transformation, indicating the oxidation of organic components within the material. The temperature range associated with Part II delineates the extent of oxidative reactions occurring within the xerogel matrix. Progressing to the final phase, Part III (Carbonization Process), the TGA curve illustrates the rapid oxidation of the CH_3_ group, resulting in a significant weight loss in the xerogel samples. At this juncture, the xerogel matrix undergoes complete carbonization. The carbonization process is pivotal, representing the decomposition of the silica xerogel structure, leaving behind only the reinforcing fibers. The temperature range within Part III signifies the culmination of thermal transformations leading to carbonization.

The segmented temperature ranges illustrated in the TGA curves (Fig 8) offer a pronounced understanding of the distinct phases of evaporation and oxidation within the xerogel samples. Crucially, the TGA results affirm the robust thermal stability of all xerogel samples, irrespective of their compositions, up to the designated maximum temperature of 600°C. Yundan Liao et.al reported delayed weigh loss in Fibre reinforced aerogel composited till 370°C which indicate better thermostability as compared to pure aerogel [21]. All TGA curve in Fig 8 displayed weigh loss after 400°C and thermal stability till 600°C that is because of high thermal stability of fibers reinforcement.

In conclusion, the synthesized xerogel samples exhibit commendable thermal stability across diverse compositions attributed to different reinforcement procedures. The average weight loss percentage, hovering around 9–10%, serves as a testament to the resilience of the material under elevated temperatures. This level of thermal stability surpasses the achievements of previous researchers [13–15], positioning the synthesized xerogels as promising candidates for applications demanding thermal resistance.

The thermal behaviour observed in the TGA analysis underscores the potential of these silica xerogel samples to withstand high temperatures, expanding their utility in various industries. The robustness displayed during evaporation, oxidation, and carbonization phases positions these materials as reliable and stable even under demanding thermal conditions. Moreover, the TGA results provide valuable insights for optimizing the synthesis process and tailoring the composition of silica xerogels for specific applications where thermal stability is a critical factor. By understanding the thermal responses at a molecular level, researchers and engineers can make informed decisions in the design and utilization of silica xerogel-based materials. In essence, this detailed thermal analysis contributes not only to the fundamental understanding of silica xerogel behaviour under heat but also paves the way for practical applications in fields ranging from insulation to aerospace, where thermal stability is paramount.

## Conclusions

Since, xerogels are capable of certain incredible properties including low density, high porosity, high mechanical strength upon reinforcement, high thermal stability, and highly hydrophobic upon surface modification make them quite suitable for fire retardants, waterproofing, and other applications. But, due to the brittle nature and high manufacturing cost, their usage is limited to a few applications. The addition of glass and carbon fiber together with silica gel particles helped in preparing flexible, less costly, hydrophobic, and crack-free silica xerogel composites by APD. In this study, TEOS-based silica xerogel composites are successfully synthesized with ambient pressure drying along with glass and carbon fiber reinforcements. Silica xerogel composites were synthesized in just 60 hours. SEM is used to reveal the structure and surface morphology of the aerogel while FTIR provides the exact chemical composition of the aerogel matrix. The compression test results give the mechanical properties of the xerogel samples and TGA provides the measure of thermal stability of the aerogel. The synthesized SX, composite SX, composite SX with GF, and composite SX with CF have a very low density of 0.1694 g/cm^3^, 0.2789 g/cm^3^, 0.1757 g/cm^3^, and 0.1732 g/cm^3^ respectively. SEM images showed that addition of fibers provide good bonding/adhesion to silica gel which give dense microstructure with less voids. Carbon fiber silica xerogel composite samples have better homogenized structure then other samples. Surface modification of the wet gel with TMCS for the whole day results in superhydrophobic structure and gives high strength to the xerogel network. However, treating for longer periods gives yellowish colour to the surface due to the presence of hydrochloric acid traces. The non-existence or a negligible peak of the OH group in FTIR graphs depicts the hydrophobic nature of the composite SX. The addition of silica gel gives high compressive strength to the composite SA by acting as a backbone to support the aerogel network. It also reduces the collapsing of the structure due to capillary pressure during the drying process by giving crack-free composite SX. Fiber reinforcements to the xerogel structure overcome its brittle nature and enhance its flexibility. Moreover, glass fiber gives a modulus compressive strength of 187.78 kPa while carbon fiber gives just 221.54 kPa. TGA curve confirm the thermal stability of aerogel samples with respect to temperatures during evaporation, oxidation, and carbonization stages. The average weight loss percentage of composite xerogels is about 9–10% by weight. Hence, the addition of glass and carbon fiber together with silica gel particles helped in preparing flexible, less costly, hydrophobic, and crack-free silica xerogel composites by APD.

## Supporting information

S1 Dataset (DOCX)
