# Supplementary material for: Investigation of mechanical and thermal behavior of fiber-reinforced silica xerogel composites
Source: PLoS One. 2024 Jun 12;19(6):e0303293. doi: 10.1371/journal.pone.0303293 (PMC11168693; doi:10.1371/journal.pone.0303293)
Supplement: S1 Dataset — (DOCX) [file pone.0303293.s001.docx]

[[
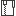
](https://uett-my.sharepoint.com/:u:/g/personal/aneela_wakeel_uettaxila_edu_pk/EV2LZoRLMVtBvG5V80ag6m8BaLAlOk57LYBZt4Z_vNVuQA)FTIR-20231129T084542Z-001.zip](https://uett-my.sharepoint.com/:u:/g/personal/aneela_wakeel_uettaxila_edu_pk/EV2LZoRLMVtBvG5V80ag6m8BaLAlOk57LYBZt4Z_vNVuQA)

[[
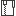
](https://uett-my.sharepoint.com/:u:/g/personal/aneela_wakeel_uettaxila_edu_pk/EWNOELL3Be9BqtKZ1stw7wcBYYDR45cXRu3GeXEXUbZvEw)SEM-20231129T084544Z-001.zip](https://uett-my.sharepoint.com/:u:/g/personal/aneela_wakeel_uettaxila_edu_pk/EWNOELL3Be9BqtKZ1stw7wcBYYDR45cXRu3GeXEXUbZvEw)
